# Impact of bilateral biopsy-detected prostate cancer on an active surveillance population

**DOI:** 10.1186/s12894-019-0452-x

**Published:** 2019-04-23

**Authors:** Jonathan H. Wang, Pablo Sierra, Kyle A. Richards, E. Jason Abel, Glen O. Allen, Tracy M. Downs, David F. Jarrard

**Affiliations:** 1 Department of Urology, University of Wisconsin School of Medicine and Public Health, Madison, WI USA; 2 Universidad CES, Medellin, Colombia; 3 University of Wisconsin Carbone Cancer Center, Madison, WI USA; 4 Wisconsin Institute for Medical Research, 1111 Highland Avenue, Madison, WI 53705-2281 USA

**Keywords:** Active surveillance, Prostate biopsy, Bilateral disease, Grade group

## Abstract

**Background:**

To assess factors that can predict active surveillance (AS) failure on serial transrectal ultrasound guided biopsies in patients with low-risk prostate cancer.

**Methods:**

We evaluated the records of 144 consecutive patients enrolled in AS between 2007 and 2014 at a single academic institution. Low risk inclusion criteria included PSA < 10 ng/ml, cT1c or cT2a, Grade Group (GG) 1, < 3 positive cores, and < 50% tumor in a single core with the majority having a PSA density of < 0.15. AS reclassification was defined as progression to GG ≥2, 3 or more cores, or core tumor volume ≥ 50%. Univariate and multivariate Cox proportional hazards regression analysis was used to determine predictors of reclassification and a match-pair analysis performed on a control group of patients choosing surgery.

**Results:**

Inclusion criteria were met by 130 men with a median follow-up of 52 months. The reclassification or AS failure rate was 38.5%, with the majority 41/50 (82%) finding GG ≥ 2 cancer. Most patients had unilateral disease on diagnostic biopsy (94.6%), but 40.7% had bilateral cancer detected during follow-up. Men with bilateral detected tumor were more likely to ultimately fail AS than patients with unilateral tumors (HR 4.089; *P* < 0.0001) and failed earlier with a reclassification-free survival of 32 vs 119 months respectively. In a matched-pair analysis using a population of 211 concurrent patients that chose radical prostatectomy rather than AS, 76% of patients with unilateral cancer on biopsy had bilateral cancer on final pathology.

**Conclusions:**

The finding of bilateral prostate cancer on biopsy is associated with earlier AS reclassification. Finding bilateral disease may not represent disease progression, but rather enhanced detection of more extensive disease highlighting the importance of confirmatory biopsy.

## Background

Currently 15% men are diagnosed with prostate cancer (PC) during their lifetime but the risk of death due to the disease is only 3% [1]. The average expected years of life lost due to PC is 1.8 years, compared to other common malignancies such as breast cancer, at 16.7 years [2]. To further emphasize this long natural history, up to 45% of patients are diagnosed with low risk PC [3].

The rationale for active surveillance (AS) is that many patients have indolent tumors and would not benefit from immediate definitive treatment. However, there is always concern from both the patient and urologist that undiagnosed, aggressive disease exists. Indeed 30–50% of patients that meet AS criteria with Gleason Score 6 cancers are upgraded at radical prostatectomy with Gleason Score pattern 4 or higher being found [4–8]. Subsequently, larger cohorts have shown that 10–38% of patients are reclassified on follow-up during AS due to Gleason Score upgrading or increased tumor volume (no. cores, maximal core involvement (MCI)) [9–14]. It is imperative to improve the ability to identify those patients at higher risk for failing AS, either due to disease progression or reclassification, in order to provide better counseling regarding treatment options and to adjust surveillance schedules. In addition, it is not clear whether or not patients that fail AS do so as a result of finding previously undetected disease or disease progression.

The objective of the study was to analyze clinical and pathologic variables available at diagnosis to determine factors that could predict reclassification and AS failure. Our hypothesis is that having a biopsy showing bilateral tumor would indicate multifocal PC, which may be associated with higher volume and higher stage disease compared to those with unilateral tumors on initial biopsy.

## Methods

### Cohort definition

A retrospective review of a previously described protocol driven cohort [15] of patients in the AS program at our institution was performed from 2007 to 2014. This was prior to the wider use of MRI for additional evaluation. Criteria utilized for AS were based on the National Comprehensive Cancer Network definition for very low risk disease including PSA < 10 ng/ml, Grade Group 1 (Gleason grade 6), ≤2 biopsy cores with cancer, and maximal core involvement (MCI) with cancer < 50%. PSA density was < 0.15 in 86% of patients, but density was not part of our exclusion criteria. Patients were followed with serial biopsies annually and delayed intervention using pre-identified clinical and pathologic cut-offs as trigger points to indicate disease progression. This study was approved by our Institutional Review Board.

Of 144 low risk patients in the AS program, 14 patients were excluded from analysis: 9 without a confirmatory biopsy, and 5 patients were T1a or T1b diagnosed after a transurethral resection of the prostate. In all patients a confirmatory biopsy was performed within 3–12 months after initial transrectal ultrasound guided (TRUS) biopsy was performed. Twelve to 16 core biopsies were obtained at each procedure. PSA was tested every 3–6 months per protocol and a follow-up biopsy was done within 1 year of diagnosis and then every 1–2 years. Patients who did not fail AS due to biopsy criteria, continued until they were reassigned to a watchful waiting (WW) non-intervention protocol due to advanced age or other significant comorbidities.

Patients were reclassified (e.g. failed AS criteria) and offered definitive therapy (radical prostatectomy or radiotherapy) if they met the following criteria on repeat biopsy: Grade Group upgrade, ≥3 cores positive for cancer, > 50% cancer core involvement or clinical progression defined as a palpable nodule greater than previously found or a change in the TNM classification.

We then performed a separate secondary analysis of 211 patients that met the same AS criteria over a similar time period (2007–2014) who declined AS and elected upfront radical prostatectomy. All pathology was read by a fellowship-trained urologic pathologist.

### Statistical methods

Kaplan-Meier method was used to estimate time to reclassification (AS failure). Univariate and multivariate Cox proportional hazards regression analysis was performed to evaluate predictors of reclassification and AS failure. All analyses were performed using software SAS 9.4 (SAS institute Inc., Cary, NC, US). Matched pair analysis was performed for the radical prostatectomy and AS populations controlling for age, PSA < BMI, Prostate Volume, and PSA Density. Statisical support was provided by Dr. Glen Leverson, PhD.

## Results

### Characteristics of patient population

The clinical features of the 130 sequential patients with low risk PC on AS after exclusions are presented in Table 1. Characteristics include a mean age at diagnosis of 61 y (range 40–75) and a follow-up from initial cancer diagnosis of 52.4 m (range 3–196) with 66% observed for more than 3 years. Among the cohort, two patients died from unrelated causes and no patients developed metastatic disease.

**Table 1:** Clinicopathologic features of patients on active surveillance at diagnosis

Variables	n (%)	Median (IQR)
Follow-up (m)	130	42.5 (24–74)
Age (years)	130	61 (57–66)
< 50 y	5 (4)	48
50–60 y	56 (43)	57
> 60 y	69 (53)	66
PSA (ng/ml)	130	5.08 (4.2–7)
< 4 ng/ml	26 (20)	3.2 l
≥ 4 ng/ml	104 (80)	5.9
PSA density	130	0.13 (0.09–0.16)
≤ 0.15	112 (86)	0.12
BMI (kg/m^2^)	130	27.5 (25.6–31.7)
≤ 30	89 (68.5)	26.2
> 30	41 (31.5)	32.8
Prostate Volume (cc)	130	42 (32–57)
< 30	23 (17.7)	24
30–60	82 (63.1)	42
> 60	25 (19.2)	73
Follow-up biopsies	130	2.5 (2–4)
2	65 (50)	2
3	29 (22.3)	3
> 4	36 (27.7)	4.5
Max Core Involvement %^a^	130	5 (3–10)

^a^Maximal Percentage of the most involved core

Prostate Specific Antigen (PSA), Body Max Index (BMI)

The majority of the cohort is staged as cT1c (126 pts., 97%), with 4 (3%) diagnosed due to an abnormal DRE (cT2a). Baseline PSA was < 4 ng/ml in 20% and median PSA was 5.08 ng/ml (0.52–9.8). Fifty-percent of the patients had 2 follow-up biopsies, 22% had 3, and 36 patients (28%) had 4 or more biopsies. At the time of analysis, 77/130 patients (59%) remained on AS with 53 patients (41%) undergoing treatment. Of this group, 16 patients chose to electively stop AS and pursue definitive treatment, rather than having treatment due to reclassification on surveillance biopsies.

### Reclassification/AS failure

Reclassification on follow-up biopsies occurred in 50/130 patients (38.5%) the majority consisting of grade progression (82%). Failure occurred solely due to the identification of cancer in > 2 cores in 9 patients, GG ≥ 2 in 12 patients and in 29 due to both. No patients were reclassified based solely on the finding of > 50% core involvement. Of our 50 AS failures, 74% (37) proceeded to treatment, and the remaining 26% [13] of reclassified patients did not receive treatment and remained on AS because of personal preference (8 patients) or switched to WW do to new comorbidities or advancing age (5 patients). Of the reclassified patients, 40% (20/50) occurred in the first 12 months, 22% during the second year and 10, 8, and 6% during years 3, 4, 5 respectively. Of note, 12% (7 pts) failed surveillance beyond 5 years.

### Reclassification according to lateralization at biopsy

**Fig. 1:**
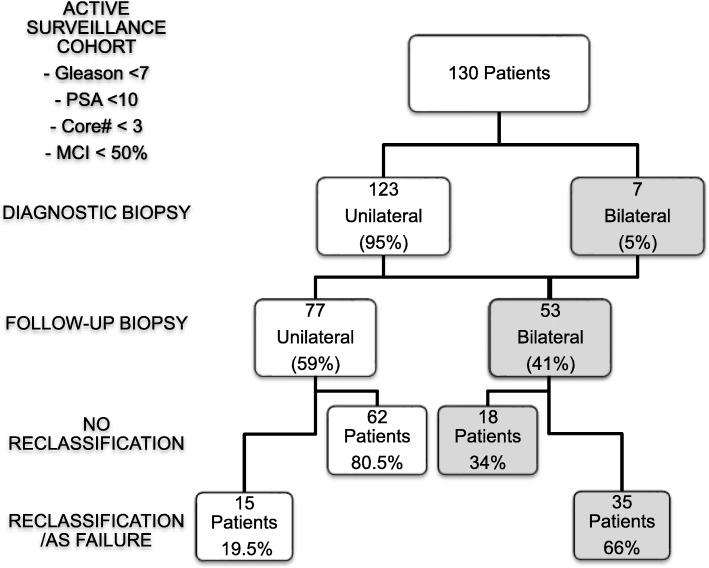
Reclassification to bilateral-biopsy detected cancer on patient follow-up. The flow chart demonstrates 41% of patients had bilateral cancer on repeated biopsies of which 66% had reclassification of their disease versus 19.5% of patients with unilateral cancer

At initial biopsy, 123 patients (94.6%) had unilateral tumor burden; the distribution for left versus right-sided disease was similar (59 and 64, respectively) and seven patients had bilateral tumor on initial biopsy (5.4%). On subsequent biopsies, 46 of the patients initially diagnosed with unilateral disease were found to have bilateral cancer. In total during AS follow-up, 77/130 patients (59.2%) continued to have tumor solely in the same lobe of the prostate on follow-up biopsies (unilateral tumor), but 53/130 (40.7%) had bilateral tumor discovered. Of these, 53 patients that initially had or developed bilateral disease, 35(66%) experienced reclassification during subsequent biopsies compared to 19.4% (15/77) those with only unilateral disease (Fig. 1). Of note, the 7 patients who initially presented with bilateral disease, 5 were reclassified during the course of our study (71%). In comparison, 29 of 46 men (63%) who initially had unilateral disease but then developed bilateral disease, while still meeting active surveillance criteria, eventually failed active surveillance.

**Fig. 2:**
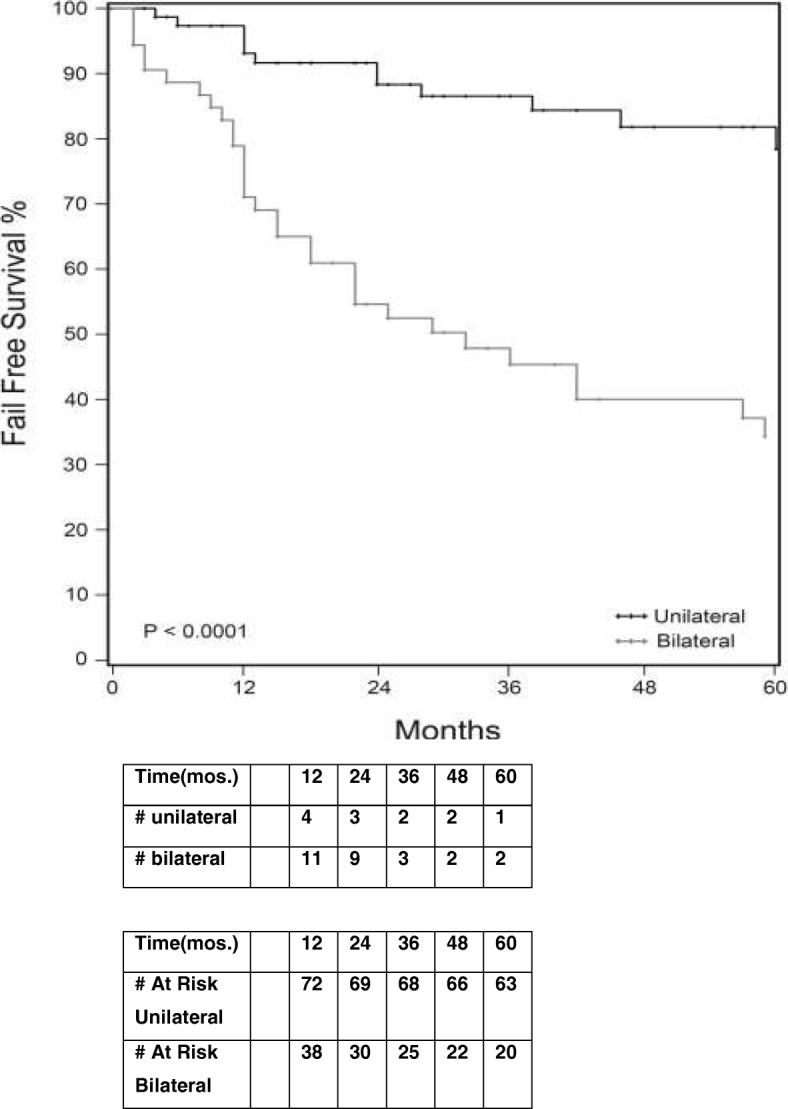
Active surveillance reclassification-free survival analysis using Kaplan Meier analysis: comparison between patients with unilateral versus bilateral cancer on prostate biopsy. Number of patients at risk shown. Patients with bilateral disease were reclassified on subsequent biopsy earlier than those with unilateral disease

On Cox proportional hazards regression analysis, patients with bilateral disease were more likely to reclassify and fail AS than patients with unilateral disease (HR 4.09; 95% CI (2.22–7.53); *P* < 0.0001) (Table 2). Patients with the finding of bilateral disease when subsequently followed over time (Kaplan Meier analysis, Fig. 2) were reclassified earlier than those with unilateral disease (32 months compared to 119 months respectively). The reclassification-free survival probability at 1 year, 2 years and 5 years was 0.71, 0.55 and 0.34 for patients with bilateral disease compared to 0.93, 0.88 and 0.78 for patients with unilateral tumor. The presence of bilateral cancer was the strongest predictor of reclassification leading to AS failure compared to all other clinicopathologic features examined.

**Table 2:** Regression analysis of clinical and pathologic features predicting active surveillance failure

Variables	Univariable COX regression			Multivariable COX regression		
	HR	95% CI	*p*-Value	HR	95% CI	*p*-Value
Bilateral tumor on Bx	4.09	2.22–7.53	< 0.0001*	3.8	2.05–7.06	< 0.0001*
MCI	1.01	0.98–1.05	0.418			
PSA	1.02	0.90–1.16	0.747			
PSA density	1.32	0.88–1.98	0.186	1.34	0.85–2.11	0.21
BMI	1	0.94–1.07	0.995			
Prostate Volume	0.99	0.97–1.00	0.131			
Previous (−) Biopsy	0.91	0.48–1.75	0.78			

Prostate Specific Antigen (PSA), Body Max Index (BMI), Maximal Percentage of the most involved core (MCI), Prostate Biopsy (Bx)

### Very low-risk patients choosing radical prostatectomy with an initial unilateral positive biopsy commonly have bilateral tumor on final specimen

We then performed an additional analysis on a separate cohort of 211 patients that met low risk, AS criteria over a similar time (2007–2014), but elected radical prostatectomy as primary treatment (Table 3). At time of diagnostic biopsy, 186 of these patients had a unilateral tumor while 25 had a bilateral tumor (88.2 and 11.8% respectively). Of the patients who had unilateral disease on diagnostic biopsy, 73.1% (136/186), were found to have pT2c (bilateral disease on final pathology) while 24.7% of the patients were found to have true unilateral disease, pT2a/b. The majority of patients who had bilateral disease on biopsy, were also found to have bilateral tumor on final pathology (88%). After surgery, 90 patients (42.6%) were upgraded on the final pathologic specimen, most being upgraded to 3 + 4 (80/90). Patients that had a bilateral tumor on biopsy did not have higher rates of positive margins at radical prostatectomy compared to patients with unilateral tumor (*p* = 0.37).

**Table 3:** Clinical and pathologic features in patients with low risk prostate cancer, eligible for active surveillance, who elected treatment with radical prostatectomy (2007–2014)

Variable	Unilateral at biopsy (*n* = 186)	Bilateral at biopsy (*n* = 25)	*p* value
Clinical Presentation	median (IQR)	median (IQR)	–
Age (years)	58 (54–64)	58 (55–61)	0.78
PSA (ng/ml)	5 (4–6.6)	4.6 (4.2–5.9)	0.51
PSAD	0.12 (0.088–0.17)	0.14 (0.1–0.18)	0.48
BMI (kg/m^2^)	29 (25.8–31.8)	28.3 (25–33)	0.86
US Vol (mL)	41.7 (32.6–53.2)	35 (25–42)	**0.03**
Max core inv.%	8 (5–20)	8 (5–10)	0.95
Grade Group	1	1	–
Stage			1
T1c	172 (92.5%)	24 (96%)	
T2a	14 (7.5%)	1 (4%)	
RP Pathology			–
Prostate weight (g)	45.4 (26–56.3)	42.5 (34.1–52.9)	0.28
Grade Group (Gleason)			0.37
GG1 (3 + 3)	106 (57%)	15 (60%)	
GG2 (3 + 4)	72 (39%)	8 (32%)	
GG3 (4 + 3)	6 (3%)	1 (4%)	
GG4 (4 + 4)	1 (0.5%)	1 (4%)	
GG5 (4 + 5)	1 (0.5%)	0	
Upgrading	80 (43%)	10 (40%)	–
% of tumor	4%	5%	0.11
Pathologic Stage			0.49
pT2a	44 (23.7%)	3 (12%)	
pT2b	2 (1.1%)	0	
pT2c	136 (73.1%)	22 (88%)	
pT3a	4 (2.1%)	0	
pT3b	0	0	

Prostate Specific Antigen (PSA), Prostate Specific Antigen Density (PSAD) Body Max Index (BMI), Radical Prostatectomy (RP)

To control for an age bias, a matched-pair analysis was performed between low risk patients who underwent surgery and the AS cohort based on age, PSA, BMI, PSA density, and prostate volume (Table 4). Comparing the 111 matched pairs, the majority of patients (75.8%) with unilateral disease at biopsy, would have had at diagnosis pT2c disease on final pathology (Tables 5 and 6).

**Table 4:** Comparison of baseline demographics at time of diagnosis between patients on active surveillance (AS) and patients who met active surveillance criteria but chose upfront radical prostatectomy (RP)

Variable	Active Surveillance	Radical Prostatectomy	
	Median (IQR)	Median (IQR)	*p*-value
Age (years)	61 (57–66)	58 (54–62)	< 0.01*
PSA (ng/ml)	5.08 (4.2–7)	5 (4–6.6)	0.39
BMI (kg/m^2^)	27.52 (25.63–31.74)	28.98 (25.78–32)	0.27
Prostate vol (mL)	42 (32–57)	40 (31–53)	0.39
PSA density	0.13 (0.09–0.16)	0.12 (0.09–0.18)	0.58

*All patients Gleason 6 at diagnosis

Prostate Specific Antigen (PSA), Body Max Index (BMI)

**Table 5:** Matched pair analysis between patients with low risk prostate cancer undergoing active surveillance or prostatectomy, controlling for age, PSA < BMI, Prostate Volume, and PSA Density. *N* = 222, (111 pairs)

Variable	Active Surveillance	Radical Prostatectomy	
	Median (IQR)	Median (IQR)	*p*-value
Age (Years)	60 (57–65)	60 (57–64)	0.9
PSA (ng/L)	5 (4.1–7)	5.3 (4.3–6.7)	0.54
BMI (kg/m^2^)	27.7 (25.7–32)	29 (26–31.2)	0.82
Prostate Volume (mL)	42 (32–57)	42 (34–56)	0.74
PSA density	0.12 (0.09–0.15)	0.12 (0.09–0.18)	0.26

**Table 6:** Biopsy results of matched pair cohort correlated with pathologic stage

Biopsy Laterality	Pathological Stage		
	pT2a	pT2c	pT3a
Bilateral n(%)	1 (6.7)	14 (93.3)	0
Unilateral n(%)	20 (21.1)	72 (75.8)	3 (3.2)

## Discussion

The selection of patients for AS according to current recommendations is guided by clinical and pathologic features that indicate the patient has a small, organ confined, well-differentiated tumor [9–14]. Patients are labeled as having failed AS if they are found to have a pathological indication to suggest that the tumor is progressing (reclassification), including increasing tumor volume (increasing number of cores with cancer or increasing volume of cancer within a biopsy core) or Gleason score upgrading [9–11, 13, 16]. Regular repeated biopsies play a pivotal role in redefining risk and reclassifying patients. Negative biopsies during surveillance follow-up occurs in 21–52% of patients and previous studies have shown these patients with no cancer on follow-up biopsies have a 53% reduction in risk of disease progression [17, 18]. There are ongoing efforts to both improve the diagnostic yield of prostate biopsies and identify factors that may predict patients who are at higher risk for failing AS in order to provide better counseling regarding treatment options and avoid unnecessary interventions.

In our AS cohort, we found that patients with bilateral tumor on initial or surveillance biopsies were reclassified during AS follow-up over time earlier and more frequently compared to those with unilateral disease (66% vs 19.5% respectively). This risk increases over time (Fig. 2). The finding of bilateral disease has a higher rate of active surveillance failure than other clinical features including PSA density. PSA density was low (< 0.15) for the majority of the population making it a less powerful indicator of failure in this population. This finding is supported by a study in which the presence of bilateral prostate cancer on biopsy was exchanged with > 50% MCI as a reason for excluding AS excusion criteria, demonstrating good performace in predicting clinically significant prostate cancer [19]. These findings suggest that the presence of bilateral disease is a clinically significant and inexpensive way to risk stratify patients enrolled in AS (Fig. 2). The presence of bilateral tumor can be used in counseling patients and as a trigger for further evaluation due to high failure rate in this group of patients.

In our analysis, bilateral disease on biopsy was the strongest parameter in predicting AS failure, outperforming other factors including the number of cores with cancer, MCI and PSA density. Other studies have assessed PSA kinetics to determine if this parameter can be used as a trigger for intervention. For example, in the PRIAS [9] study, the largest ongoing prospective study, PSA doubling time < 3y was a trigger for intervention. However, Klotz et al. [11] discontinued its use in 2009 and is not being used in other larger cohorts such as the John Hopkins AS group [11] or UCSF. There is also suggestive data that PSA density can be a strong predictor for reclassification over longer term time periods, however more studies are needed to confirm these findings [9, 20, 21]. In our data PSA density did not perform as well as the detection of bilateral cancer likely due to the fact that the majority (86%) of patients had a PSA density of < 0.15 and thus only a limited range was evaluated.

An important question is whether patients fail AS due to disease progression or due to detection at subsequent biopsy. Conceptually TRUS biopsy, although templated, is a procedure with a relatively low negative predictive value. In our separate analysis of a concurrent cohort of 211 patients eligible for AS but elected for upfront radical prostatectomy, 73% of these patients had bilateral, pT2c disease on final pathology. This was confirmed with our matched pair analysis. Studies have demonstrated that 71–80% of low risk patients have bilateral tumor on final specimen when taken for upfront radical prostatectomy [22–24]. This suggests that the majority of the patients with PC have multifocal, bilateral tumors that are missed during TRUS biopsy even in a selected low risk population.

While it is important to fine tune the ability of urologists to use standard TRUS biopsy information to guide treatment counseling, MRI increasingly is being employed. In our active surveillance cohort, 42/130 (32%) patients had a prostate MRI either prior to diagnosis of prostate cancer, or as part of their follow-up. Recent improvements in MRI technology increase the yield of prostate biopsy, but not all practitioners worldwide have access to high quality imaging. To illustrate the limits of access to this technology, Japan has the most MRI units per million population (47) with the US second (38 upm). This is compared to countries like Germany (12 upm), France (11 upm), Canada (9 upm), UK (6 upm), and Mexico (2 upm) [25]. MRI has a lower sensitivity of 63% in detecting lower volume (< 0.5 ml) and intermediate grade (Gleason 7) PC, but does have up to 80% detection rate for higher grade Gleason > 8 PC [26]. There has been an increased use of MRI during AS to help determine eligibility and disease progression [27]. These technologies are not yet widely adopted due to cost and access especially in underserved populations. MRI guided, targeted prostate biopsies may improve our management of AS patients and could be important in driving changes in biopsy schedules or AS criteria with additional data. With limited MRI access, these data demonstrating greater risk with bilateral positive biopsies may help direct those patients who need further radiologic evaluation.

Our AS failure rate is 38.5% is slightly higher compared to other cohorts as reported by Klotz et al. [11], PRIAS [9], Tosoian et al. [10], Preston et al. [12], Dall’Era et al. [13], (22.6, 28, 30.6, 34.7, 38%). These variations in failure rates may result from different criteria or the population utilized for the study. Our study is limited in its retrospective nature and single institution, but it is strengthened by our assessment of a concurrent group of patients who met AS criteria and elected definitive treatment. A matched-pair analysis was performed in an effort to limit treatment bias in this population.

## Conclusions

Finding bilateral PC on TRUS biopsy is an important predictor of reclassification or AS failure. Our data supports the use of identification of bilateral disease on prostate biopsy to guide patient counseling on AS, as these patients are more likely to require reevaluation with MRI or repeat biopsy at an earlier time. Given the high rate of bilateral PC seen in a concurrent low risk AS-eligible population with unilaterally positive biopsies, the development of bilateral disease represents improved detection rather than progression for many of these patients.

## Abbreviations

AS: Active Surveillance
GG: Grade Group
MCI: Maximal core involvement
PC: Prostate Cancer
PSA: Prostate Specific Antigen
